# Correlation and agreement between infrared thermography and a thermometer for equine body temperature measurements

**DOI:** 10.14202/vetworld.2023.2464-2470

**Published:** 2023-12-20

**Authors:** Kannika Na Lampang, Ashannut Isawirodom, Porrakote Rungsri

**Affiliations:** 1 Department of Veterinary Biosciences and Veterinary Public Health, Chiang Mai University, Chaing Mai, 50200, Thailand; 2 Faculty of Veterinary Medicine, Chiang Mai University, Chaing Mai, 50200, Thailand; 3 Department of Companion Animal and Wildlife Clinic, Chiang Mai University, Chaing Mai, 50200, Thailand

**Keywords:** body temperature, digital thermometer, horse, infrared thermography, radiation

## Abstract

**Background and Aim:**

Body temperature is a vital sign that determines physical status. Infrared thermography (IRT) is more frequently used for assessing horses’ temperature because of its ease of use and less contact with the horses, making it a safer measurement method. However, the accuracy of IRT remains unclear; therefore, this study aimed to assess the potential use of IRT as an alternative method for measuring horse body temperature.

**Materials and Methods:**

Temperatures were measured in 14 horses. A digital thermometer was used to collect rectal temperature (RT), whereas a thermographic camera was used for IRT at three different positions to obtain the center of body temperature (CBT), head temperature (HT), and eye temperature (ET). The protocol was performed over 30 days, repeated thrice daily: morning (6:00–8:00), afternoon (14:00–15:00), and evening (17:00–19:00). Environmental factors, including humidity, ambient temperature, wind flow, and light intensity, were recorded indirectly according to the time of day and cooling device use.

**Results:**

Mean RT, CBT, HT, and ET were 37.33°C, 34.08°C, 35.02°C, and 35.14°C, respectively. Center of body temperature was lower than RT by an average of 3.24°C (95% confidence interval [CI], 5.4°C–1.09°C). HT was lower than RT by an average of 2.3°C (95% CI, 4.33–0.28). The eye position showed the least difference between RT and infrared temperature, with an average of 2°C (95% CI, 0.7–3.92). However, there was no significant correlation between RT and infrared temperature at any position. Spray and vaporizer use significantly affected IRT and time of day (p = 0.05).

**Conclusion:**

Although IRT has advantages in terms of non-invasiveness and reduced stress on horses, its accuracy and reliability may be compromised by environmental variables, which interfere with infrared measurement. Future research should specifically focus on investigating environmental factors.

## Introduction

Horse owners routinely monitor their animals’ body temperature to prevent heat-related issues for medical purposes and during athletic activities [1, 2]. Traditional rectal thermometers are commonly used to measure core body temperature; however, they can be intrusive and difficult to use, especially for untrained or agitated horses [3]. Therefore, alternative tools, such as thermal microchips, infrared thermometers, and infrared cameras, have been developed. However, the temperature recorded by the microchip does not correspond to the rectal temperature (RT); instead, it reflects the temperature of the skin surface where the microchip is implanted [4]. In contrast, infrared devices operate on the principle of body heat dissipation and are widely used in the medical field that involves the following four mechanisms: Radiation, convection, conduction, and evaporation. Radiation, a key heat dissipation component, primarily occurs in the infrared frequency range [5, 6]. In humans and veterinary medicine, tympanic temperature is measured by infrared thermometers [3, 4]. However, it can be challenging with horses because of the need for ear insertion [4]. Thermographic cameras, which convert infrared waves into visual images, are another temperature measurement tool. Infrared thermography (IRT) has recently become popular in assessing conditions such as back pain, lameness, laminitis, and stress [7, 8].

Equine IRT is gaining traction for many reasons. It is non-invasive and thus reduces stress on horses. In addition, IRT allows measurements to be taken from a distance, which minimizes the risk of accidents during rectal thermometer insertion, especially in untrained or excited horses [3]. Its simplicity and ease of use make it an interesting alternative for equine temperature assessment. Previous studies [9, 10] have used infrared spectroscopy (IRT) to measure body temperature in various parts of the equines’ bodies, focusing primarily on the head and body axis to determine the most accurate infrared temperature to express changes in the core body temperature. The reliability and non-reliability of IRT in the body trunk have been demonstrated [9, 10]. On the other hand, the head is preferable, especially the eye region, because it is abundant in blood supply, and the skin is thinner than other parts of the body. However, there is a noticeable difference in the study results. In 2011, a study the 24 ponies reported that infrared eye temperature (ET) is related to RT and could be used as an additional technique for body temperature screening [11]. In contrast, Jansson *et al*. [12] found no relevance between ET and RT and suggested using the same conventional method.

By adopting IRT, horse owners and veterinarians may have a valuable tool for monitoring and assessing equine body temperature without requiring invasive procedures. Infrared thermography enables temperature measurements to be collected from a distance, thereby reducing the risk of accidents during rectal thermometer insertion. However, the suitability of thermography for body temperature measurement remains unclear. Therefore, this study aimed to assess the feasibility and accuracy of IRT as an alternative method for measuring equine body temperature.

## Materials and Methods

### Ethical approval

This study was approved by the Ethics Committee of the Faculty of Veterinary Medicine, Chiang Mai University Animal Care and Use Center. The approval code was FAC-ACUC; S29/2562.

### Study period and location

This study was conducted from June to September 2019 in Chiang Mai, Thailand. The ambient temperature ranged from 23°C to 43°C, and the relative humidity ranged from 18% to 40.5%.

### Animals

This study included 14 mixed-breed sport horses: One stallion, nine geldings, and four mares. Among these, four horses were used for schooling, five were in dressage, two were show jumping horses, and three were retired. The ages of the horses ranged from 8–20 years; their body condition scores were estimated as 3/5 using the Caroll and Huntington body condition scoring method [13]. All horses were in good health throughout the study.

### Temperature measurement

A digital thermometer (Omron-245, Omron Healthcare Co., Ltd., Japan) was used to measure RT. This thermometer has been designed to provide an alarm within 1 min of pressing the power button, ensuring quick and accurate temperature readings. The infrared temperature was measured using a portable thermographic camera (FLIR C3, FLIR Systemic, Inc., Oregon, USA). This camera is approximately the size of a mobile phone and provides infrared images with temperature indications. The infrared images’ temperature values were calculated by considering factors such as emissivity and the distance between the camera and the object. It is important to note that both of these parameters can be manually adjusted for precise measurements.

Before the measurements, the horses were positioned and held against the wall with their right sides. The digital thermometer was inserted into the horse’s rectum for 1.5–2 inches with the tip of the thermometer coming in contact with the dorsal area of the rectum, and the temperature was read when the tool finished measuring.

**Figure-1:**
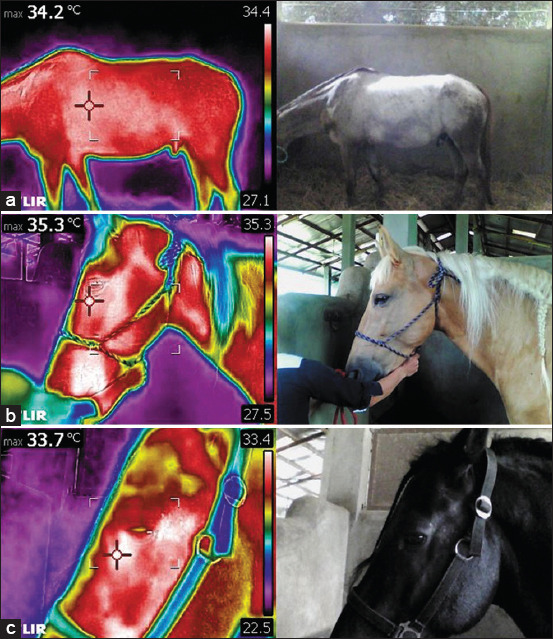
Thermography positions present in infrared image and normal image, including (a) center of the body, (b) head and (c) eye.

IRT on the left side of the horses was performed by only one examiner throughout the study. Three positions were captured, namely, the center of the body, head, and eye, as shown in Figure-1. The camera parameters were set as follows: emissivity at 1.00; distance was set following the position where the image was taken, which was 3 m for the center of the body, 1 m for the head, and 0.5 m for the eye. The distance from the camera to specific positions was determined based on a known stall length of 3.5 m. As the horse’s right side was held against the wall, the examiner could stand on the opposite side of the wall to capture center of body temperature (CBT). Head temperature (HT) measurements were collected at a distance of 1 m from the horse’s head, aligned with the stall door. To ensure accurate readings, the camera was held perpendicular to each position. The thermographic camera was calibrated to capture the temperature of each horse before each use. The highest temperature was recorded in the left corner of each image.

Temperatures were recorded when the horses rested in their own stalls, before exercise or after exercise, and cooling for at least 2 h. We performed the protocol on all 14 horses for 30 days and was repeated thrice daily at different periods, including the morning (6:00–8:00), afternoon (14:00–15:00), and evening (17:00–19:00). These are the three periods were set to cover different environmental variables in 1 day, aligning with farm staff’s working hours. Eye temperature was recorded for only 2 days because most of the temperature readings from the head position primarily focused on the eye area because the eye position was added to the protocol.

Environmental factors such as ambient temperature, light intensity, humidity (spray and vaporizer), and wind flow (fan) in the stall were indirectly presented by the time of day and the cooling devices. The cooling device used was recorded at the time of measurement.

### Statistical analysis

Data were analyzed using R version 4.2.3 (R program, version 4.2.3, The R Foundation, Vienna, Austria) and R studio version 3.5.3 (R studio, Posit, Boston, MA, USA). Body temperatures measured using a thermometer and a thermographic camera were examined using linear regression to determine correlations. Bland-Altman analysis was used to determine the agreement between each method, which comprised between devices (thermometer and thermographic camera) and between thermographic positions (center of body and head).

Potential environmental effects, such as ambient temperature, humidity, light intensity, and wind flow, that can interfere with heat distribution and increase the IRT error were considered. However, because of the lack of direct data, the time of day was used to present a wide range of ambient temperature and light intensity, and the effect of wind flow (fan) and humidity (spray and vaporizer) as indirect indicators were determined. Time of day (morning, afternoon, and evening) and cooling devices (fan, vaporizer, and spray) as independent variables were analyzed using repeated analysis of variance with temperature as the dependent variable. All statistical analyses were considered statistically significant at p *=* 0.05.

## Results

**Figure-2:**
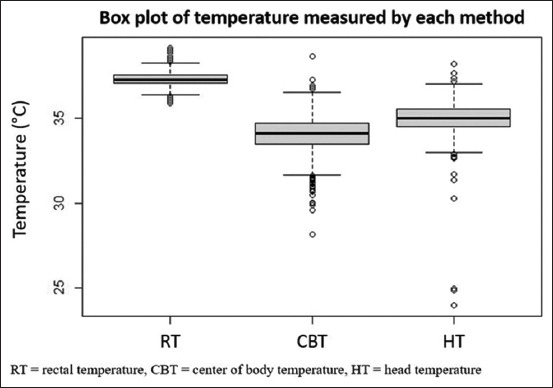
Comparison of temperature obtained by different methods: Rectal temperature range is 35.90–39.20 (interquartile range [IQR] = 0.5), center of body temperature range is 28.13–38.70 (IQR = 1.23), and head temperature range is 23.93–38.23 (IQR = 1.03).

One thousand three hundred and fifty-four records were obtained from 14 horses; each record contained the horse’s ID, RT, CBT, HT, time of temperature measurement, and cooling devices used. Figure-2 shows the comparison among RT, CBT, and HT.

### Agreement and correlation between the methods

**Figure-3:**
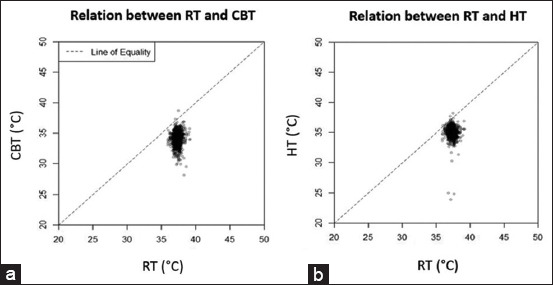
Correlation between temperatures measured by different methods, (a) correlation between rectal temperature and center of the body temperature, and (b) correlation between rectal temperature and head temperature.

No significant correlation was observed between RT and CBT, but a statistically weak negative correlation was observed between RT and HT (coefficients = 0.05, p = 0.01). This indicates that the thermographic camera is inconsistently aligned with the thermometer, potentially leading to incorrect interpretation of body temperature. Most thermographic camera temperatures tend to underestimate body temperature, as shown in Figure-3.

**Figure-4:**
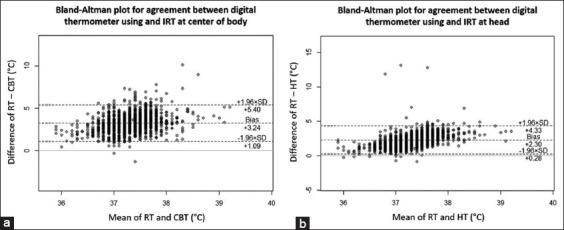
Bland-Altman plot of agreement between two methods, X-axis represents mean of rectal temperature (RT) and head temperature (HT), and Y-axis represents difference between two methods: (a) Agreement between digital thermometer using and infrared thermography (IRT) at center of the body, and (b) agreement between digital thermometer using and IRT at head. Each grey dot represents one record of data, increasing in density mean more overlapped data. CBT=Center of body temperature.

The mean RT was 37.33°C, and the mean CBT and HT were 34.08°C and 35.02°C, respectively. Under ideal conditions, both techniques should be indifferent and all data should fall within an agreement limit of 1.96 standard deviation (SD) [14]. Analysis of agreement between RT and CBT showed an average difference of 3.24°C with a limit of agreement (95% confidence interval [CI]) between 5.40 and +1.09. Within-subject SD (Sw) and repeatability coefficient (RC) were 2.42 and 6.71, respectively (difference between the upper and lower limits). A lesser difference was found between RT and HT at 2.30°C with a narrower limit of agreement (95% CI) from 4.33 to +0.28. The Sw and RC were 1.78 and 4.95, respectively, as shown in Figure-4.

### Agreement and correlation between IRT positions

**Figure-5:**
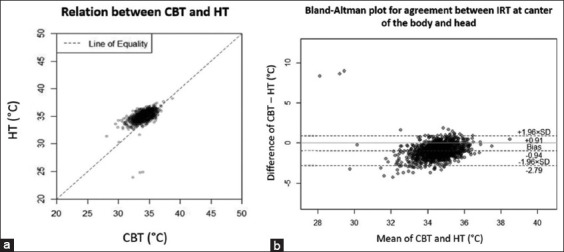
Correlation and agreement between center of the body temperature (CBT) and head temperature (HT). (a) Correlation between CBT and HT. (b); Bland-Altman plot of agreement between CBT and HT, X-axis represents mean of two infrared temperatures and Y-axis represents difference of two temperatures.

There was a good correlation between CBT and HT (coefficient = 0.61, p = 0.01). In addition, we evaluated the agreement between CBT and HT. The average difference between CBT and HT was 0.94°C and the limit of agreement ranged from 0.91 to 2.79. Within-subject SD and RC were 0.94 and 2.61, respectively. Differences in position created a fair amount of change in temperature, but it was smaller than that observed between devices, as shown in Figures-5.

### Eye temperature

**Figure-6:**
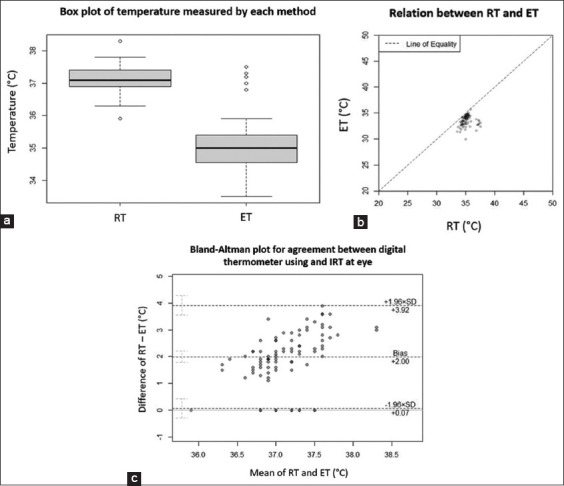
Eye temperature (ET) analysis. (a) Box plot shows ET and rectal temperature (RT), the RT range from 35.90–38.30 (interquartile range [IQR] = 0.5) and ET range from 33.5–37.50 (IQR = 0.85). (b) Correlation between RT and ET. (c) Bland-Altman plot of agreement between RT and ET. X-axis represents mean of two temperatures and Y-axis represents difference of two temperatures.

A separate analysis of eye position was conducted because only 87 records contained ET. Rectal temperature ranged from 35.90 to 38.30 (interquartile range [IQR] = 0.5) and ET ranged from 33.5 to 37.50 (IQR = 0.85) in these records. No significant correlations were found between these temperatures (adjusted R^2^ < 0.01, p = 0.70). The Bland–Altman plot shows the mean RT and ET at 37.13°C and 35.14°C, respectively, with an average difference of 2°C. The limit of agreement ranged from 0.07 to 3.92. The Sw and RC were 1.57 and 4.3. The analysis results are shown in Figure-6.

### Potential environmental factors

The period and cooling devices used to collect the temperature were noted and analyzed to determine whether factors interfered with the thermography technique. One thousand two hundred and seventeen records were included in this analysis. The period was divided as follows: into the following three categories: Morning (6:00–8:00), afternoon (14:00–15:00), and evening (17:00–19:00 There was a significant difference between the morning and the two other periods, p *=* 0.05. In the morning, there was an approximately 0.5°C increase in the significant difference, p = 0.05. Table-1 shows differences between the conventional method and IRT at each period.

**Table-1:** The difference between the temperature measured with a digital thermometer and a thermographic camera in each time period is presented in mean ± SD.

Time	Difference between rectal temperature and infrared temperature at each position	

	Body position	Head position
Morning	2.59 ± 0.91*	2.58 ± 0.75*
Noon	2.1 ± 0.80	2.12 ± 0.79
Evening	2.16 ± 0.79	2.19 ± 0.90

* Significant different from other time periods within column (p < 0.05)

Analysis of cooling devices included 1,314 records. It comprises a fan, vaporizer, and spray. The results showed that humidity or water droplets may have interfered with the thermographic camera. No significant interaction was observed between the temperature difference measured by XRD in the two devices and the fan used. However, there was a significant difference in the presence of the vaporizer (p *=* 0.05), similar to the spray, as shown in Table-2. For CBT, the presence of the vaporizer reduced the temperature by approximately 0.5°C, whereas the spray reduced it by approximately 0.3°C, with both having a significance level of p *=* 0.05. The spray also reduced HT by approximately 0.3°C (p = 0.05).

**Table-2:** Mean ± SD of difference in temperature between thermometer and thermographic camera in the present of cooling devices.

Cooling devices	Difference (body)	Difference (head)
Vaporizer (with)	2.70 ± 0.748	2.60 ± 0.748
Vaporizer (without)	3.21 ± 0.979	2.25 ± 0.865
Spray (with)	2.97 ± 0.953	2.21 ± 0.812^a^
Spray (without)	3.26 ± 0.974	2.28 ± 0.877^a^

a No difference between with and without (p > 0.05)

## Discussion

In this study, statistical analysis revealed no correlation between the temperature obtained from the thermometer and the temperature captured by the thermographic camera at the center of the body. In addition, there was a very low negative correlation between HT and RT.

A recent study showed no correlation between RT and measured infrared temperatures from the five regions from the neck to the croup of horses [10]. In a previous study by Giannetto *et al*. [15] in cats, the same outcome was found when other areas of the body axis were used. Both studies supported the use of skin temperature to evaluate inflammation in various body areas but did not determine the core body temperature.

Conversely, the results showed that the head position provided a higher mean temperature, which was closer to RT than the body position. Furthermore, the HT deviation was smaller than that of the CBT. Mostly, the hottest areas on the head on which the thermographic camera focused were around the orbital region and the upper part of the jugular groove. There were only 87 records that particularly contained ET. The eye region gave a smaller temperature difference to RT than the other two positions, but after analysis, neither a correlation nor a significant agreement was found between RT and ET.

The correlation between ET and RT varied in other studies. For example, in 2021, 32 horses of various breeds were used to assess the relationship between ET measured using IRT and RT collected using the conventional method. The authors concluded that ET had no correlation with RT [12]. In contrast, another study on Welsh ponies [11] and Italian saddle horses [16] considered ET a reliable temperature that correlated well with RT. However, the horses in each study were different in breed and sex, as was the environment where the temperature was collected; therefore, these factors might interfere with the results, as reported by Jansson *et al*. [12].

A study on koalas was conducted to determine the accuracy of IRT, and it was found that the most reliable location on the body was the eye because it is related to the body temperature of the animals [17]. Similarly, Giannetto *et al*. [18] reported that ET could be used instead of RT in cats. On the other hand, Farrar *et al*. [19] conducted research on swine to determine the relationship and agreement between RT and infrared temperature capture at two different positions the eye and the base of the ear. This finding demonstrated a relationship and an agreement with neck infrared temperature, whereas only an agreement was found for ET.

Blood vessel density, muscle mass, skin and fat thickness, and hair length are the major components of infrared radiation. According to heat dissipation processes, the heat inside the body is transferred to the skin surface through blood vessels before being emitted, whereas muscle plays a main role in heat generation, whereas fat has thermal insulation ability to retain heat inside the body. Because the IRT provides the surface temperature, which depends on the following three compositions of each area, different temperatures were displayed in the thermographic image at each part. The ocular and jugular regions of the horse’s head have more prominence in blood vessels because the facial skin has thinner fat and shorter hair than the body site, allowing for more effective heat radiation.

There was a large difference in output between the thermometer and thermographic camera, which may have been caused by uncontrolled environmental variables. Light and humidity might affect IRT. In view of the lack of direct data on brightness and humidity, we used the time at measurement and the presence of cooling devices instead.

**Figure-7:**
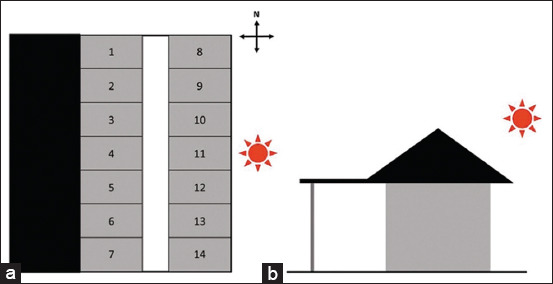
Stable diagram viewing from different aspect. (a) Dorsal aspect of stable, there are fourteen horse stalls which divided into two sides along the stable, seven stalls per each. (b) Frontal aspect of stable. Black structures demonstrate roof area.

We expected the temperature difference to be greatest in the afternoon when the ambient temperature was highest and the sunlight was strongest. However, the results showed that the temperature difference was substantially larger in the morning than in the afternoon and evening. It was assumed that this is because of the location of the stable. For clarity, a stable is an open-air building arranged from south to north and covered with a roof longer to the west than to the east. There were 14 horse stalls along both the East and West sides of the building as shown in Figure-7. In the morning, the horses living on the east side will receive the sunrise, which will shine directly on them, so half of the infrared temperature will be affected by light intensity. On the other hand, in the afternoon and evening, when the sun moves to the west, all horses will be shaded, so there will be fewer light effects in these periods. An infrared picture should be taken at low light intensity to use a thermographic camera effectively to avoid exogenous radiant energy [7].

In addition, the temperature gradient between the objects is associated with thermal radiation [20]. Yanmaz *et al*. [7] recommended performing IRT at an ambient temperature below 30°C because high temperatures indicate high radiation from environmental objects that can interfere with IRT.

In addition, humidity is involved in the thermographic imaging. The vaporizer made a considerable difference between the RT and infrared temperatures in CBT and HT, and the spray made a significant difference in CBT. There was no statistical difference in RT and HT when the spray was used because the spray was placed at the back of the stall, whereas the horses’ heads pointed toward the front during the temperature measurement. According to Hou and Tsai [21], an increased relative humidity can decrease the ability of a radiant measuring tool. This is because vapor and water spray in the air absorb heat energy from thermal radiation, decreasing the amount of heat detected by the thermographic camera, and resulting in a higher difference between the RT and infrared temperature. Alternatively, the water droplet from the spray can attach to the horse’s skin surface and enhance the evaporation heat loss mechanism instead of radiation, resulting in less infrared emission captured by the camera.

## Conclusion

There was insufficient agreement between the use of a thermometer and IRT. Therefore, IRT alone is not recommended for use in medical settings to measure body temperature. However, it may be useful as an additional tool in addition to standard techniques to provide greater accuracy and details when monitoring or predicting body temperature changes. Environmental factors such as light and humidity can significantly affect IRT results. Techniques and processes need to be modified to increase accuracy and minimize the influence of external factors. If IRT is used, it is recommended to ensure a clear background, minimal reflection, and direct exposure to light sources. In addition, the environment must be free of water droplets or any substances or objects with a significant temperature difference compared to the surrounding environment. In future research, environmental indicators, including ambient temperature, light intensity, and humidity, should be recorded separately for each measurement.

## Authors’ Contributions

KNL: Formal analysis, data curation, writing-reviewing and editing, and statistical analysis. AI: Methodology, investigation, data curation, and writing-original draft. PR: Conceptualization, methodology, resources, writing-reviewing and editing, supervision, project administration. All authors have read, reviewed, and approved the final manuscript.
